# A comprehensive analysis of the KLRB1 expression and its clinical implication in testicular germ cell tumors: A review

**DOI:** 10.1097/MD.0000000000037688

**Published:** 2024-04-12

**Authors:** Luyu Li, Yaorui Hu, Xiao Li, Baojun Ju

**Affiliations:** a The First Clinical School of Medicine Henan University of Chinese Medicine, Zhengzhou, Henan 450000, China; b Department of Neurobiology, Cheeloo College of Medicine, Shandong University, Jinan, Shandong 250012, China; c Institute of Neurobiology, Health and Rehabilitation Sciences of University, Qingdao, Shandong 266000, China; d Department of Andrology, the First Affiliated Hospital of Henan University of Chinese Medicine, Zhengzhou, Henan 450000, China.

**Keywords:** immune infiltration, KLRB1, prognosis, testicular germ cell tumors

## Abstract

Testicular germ cell tumors (TGCT) are the most common testicular malignancies. KLRB1 is considered to influence the development and progression of a number of cancers. However, it is unclear how the KLRB1 gene functions in TGCT. First, it was determined the expression level of KLRB1 in TGCT using The Cancer Genome Atlas (TCGA) (The Cancer Genome Atlas) dataset and GTEx (Genotype-Tissue Expression) dataset. The clinical significance and biological functions of KLRB1 were explored using the TCGA dataset, and we analyzed the correlation of the KLRB1 gene with tumor immunity and infiltrating immune cells using gene set variation analysis and the TIMER database. We found that the expression level of KLRB1 was upregulated in TGCT malignant tissues with the corresponding normal tissues as controls, and KLRB1 expression correlated with clinicopathologic features of TGCT. Functional enrichment analysis suggested that KLRB1 might be involved in immune response and inflammatory response. KLRB1 was highly positively correlated with natural killer cell activation in immune response and positively correlated with tumor-infiltrating immune cells. This study demonstrated for the first time the role of KLRB1 in TGCT, which may serve as a new biomarker associated with immune infiltration and provide a potential therapeutic target for the treatment of TGCT.

## 1. Introduction

Testicular germ cell tumors (TGCT) are rare malignancies which affect young adults and adolescents between the ages of 15 and 40 years. TGCT accounts for approximately 1% of all solid tumors and more than 95% of all testicular malignancies, and its incidence has continued to increase globally in recent decades with a significantly higher burden in the future.^[1,2]^ TGCT includes both germ cell neoplasias in situ (GCNIS) and non-GCNIS spermatogonial tumors.^[3]^ GCNIS includes seminoma and nonseminoma (NSE), and NSE can be subdivided into embryonal carcinoma, teratoma, chorionic epithelial carcinoma, etc.^[4]^ The main treatment for TGCT is chemotherapy represented by platinum-based drugs, which can significantly reduce the mortality of TGCT.^[5]^ However, there is still a lack of effective treatment for platinum-refractory TGCT. In addition, surgical treatments, including radical orchiectomy, as well as adjuvant treatments such as radiotherapy and chemotherapy, can seriously affect the physical and mental health of patients, leading to sexual dysfunction, infertility, cardiovascular disease, and other adverse consequences.^[6]^ It has been shown that there is a marked difference in the distribution of immune cells in TGCT, compared to normal testes or inflammatory lesions associated with hypospadias.^[7]^ Currently, cancer immunotherapy offers a new modality cancer treatment and is being considered for full application in TGCT.^[8]^ Unfortunately, however, the results of published IC inhibitor monotherapy studies to date appear to be disappointing, and we currently still lack valid predictors of clinical decision-making in routine practice.^[9,10]^

Killer cell lectin-like receptor subfamily B member 1 (KLRB1), which encodes the inhibitory T-cell receptor called CD161, plays a crucial role in tumorigenesis development and tumor immunity,^[11–13]^ and may be a prognostic and immune biomarker across tumors.^[14]^ KLRB1 may synergize with other ICs to regulate the immune microenvironment and could be used to develop new targeted immunotherapeutic agents.^[15]^ Currently, KLRB1 has shown potential biomarker value and immunomodulatory effects in breast cancer, esophageal squamous cell carcinoma, and glioma.^[16–18]^ In addition, KLRB1 enhances the progression and evolution of gliomas through its unique effect on T-cell dysfunction.^[18]^ However, the correlation between KLRB1 expression in TGCT and its biological function is unclear.

In this study, we first evaluated the association between KLRB1 expression and clinicopathology in patients with TGCT using TCGA (The Cancer Genome Atlas) and GTEx (Genotype-Tissue Expression) databases. At last, our results reveal the expression level and potential biological functions of KLRB1 in TGCT and provide a new and clearer understanding of the relationship between KLRB1 in tumor immune cells

## 2. Materials and methods

### 2.1. Public data collection and analysis

The RNA sequencing data and corresponding clinical information for TGCT patients were obtained from the TCGA data portal (https://tcga-data.nci.nih.gov/tcga/). To minimize statistical bias in the analysis, we excluded TGCT patients with missing overall survival (OS) values. Thus, we obtained a TCGA dataset consisting of 134 patients. We utilized the Genotypic Tissue Expression (GTEx) database (https://www.gtexportal.org/home/-index.html) to obtain KLRB1 gene expression data in 361 normal tissues. In addition, we focused on the expression levels of the KLRB1 gene in a variety of cancers and normal tissues using the combined data from the TCGA and GTEx databases. Moreover, the distribution and subcellular localization of KLRB1, as well as the expression in TGCT were observed by Immunohistochemistry (IHC) images using the Human Protein Atlas (THPA) (https://www.proteinatlas.org/).

### 2.2. Functional enrichment analysis

Pearson correlation was performed using RStudio (R 4.3.1). The most relevant genes of KLRB1, or a characteristic gene list of the cell cluster, were uploaded to the Database for Annotation, Visualization, and Integrated Discovery (DAVID, v6.8). The official gene symbol was selected as an identifier, and Homo sapiens was selected as the species. Finally, Gene Ontology (GO) analysis and Kyoto Encyclopedia of Genes and Genomes (KEGG) pathway analysis enrichment results were obtained. The top 7 results in ascending order of *P*-value (*P* < .05) were displayed in this study.

### 2.3. Gene set variation analysis (GSVA)

The list of genes for the immunization process was obtained from the AmiGO 2 portal (http://amigo.geneontology.org/amigo). Functional enrichment scores were calculated for each testicular cancer sample using the given package (R environment) under default parameters. The enrichment results were plotted as heatmaps using the pheatmap package (R environment), and the correlation between KLRB1 and immune processes was determined by Pearson correlation analysis.

### 2.4. Protein–protein interaction analysis

We investigated protein–protein interactions of KLRB1-binding proteins through the STRING website (https://string-db.org/), an online search tool for analyzing known proteins and predicting PPI networks, including direct and indirect interactions between proteins and their functional relevance (https://string-db.org/).^[19]^ Then, the interaction networks of KLRB1 binding proteins identified with experimental evidence were obtained. Jvenn is an interactive Venn diagram viewer used to perform cross-tabulation analyses for comparing genes related to KLRB1 expression with KLRB1-interacting genes.^[20]^ Correlations between KLRB1 expression and common genes in the crossover analysis were characterized by Spearman correlation analysis. *P* < .05 was defined as statistically significant.

### 2.5. Correlations between KLRB1 expression and immune characteristics

TIMER2.0 (Tumor immune estimation resource, version 2), a comprehensive resource database, is designed for systematically analyzing the infiltration of 6 immune cells, including B cells, CD4+ T-cells, CD8+ T-cells, neutrophils, macrophages, and myeloid dendritic cells across diverse cancer types (http://timer.cistrome.org/).^[21]^ We then used the “Gene Module” and “SCNA Module” on the website to examine the correlation between immune infiltration and the expression of KLRB1 in TGCT patients.

### 2.6. Statistical analysis

A descriptive analysis of TGCT patients was conducted for clinicopathological features. The *t* test was adopted to compare the different expression levels of KLRB1 in tumor tissues and normal tissues. All R packages were performed using R software version v4.3.1, and a level of *P* < .05 was defined as statistical significance.

## 3. Results

### 3.1. The mRNA expression level of KLRB1 was upregulated in TGCT

**Figure 1.**
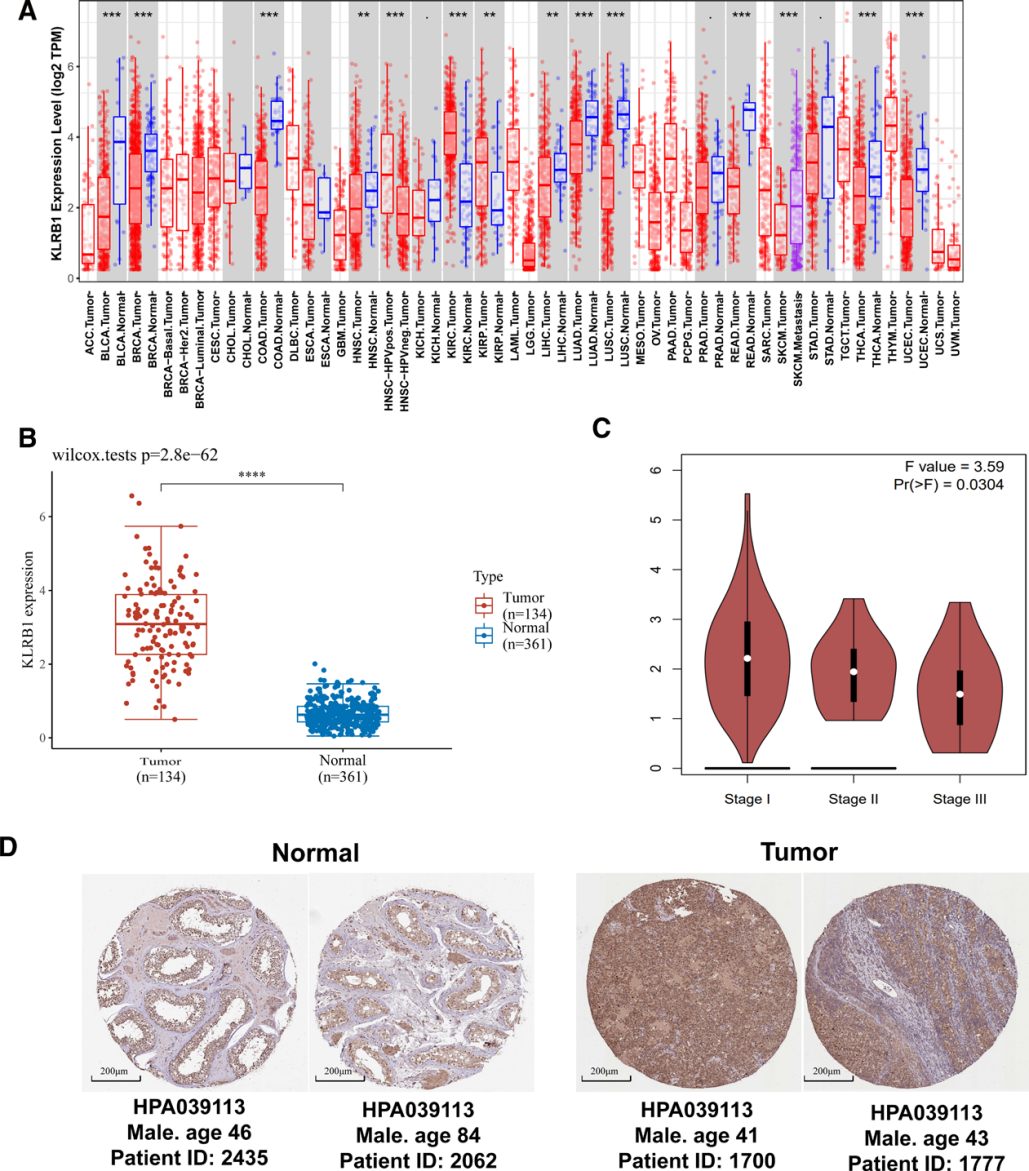
The expression level of KLRB1 gene in TGCT patients. (A) KLRB1 expression in different cancer types based on the TCGA database. (B) KLRB1 expression in normal and tumor tissues based on TCGA and GTEx databases. (C) KLRB1 expression were analyzed in different pathological stages (stage I, stage II, and stage III) based on the TCGA database. (D) Representative immunohistochemistry images and detailed information on the expression of KLRB1 in TGCT tumor tissues and normal tissues based on the THPA database. *P < .05, **P < .01, and ***P < .001.

To compare KLRB1 expression in human cancers, we used the TCGA database to assess how KLRB1 is expressed in multiple types of cancers (Fig. 1A). Next, considering the limited adjacent normal tissues in TCGA database, we also compared the expression level of KLRB1 gene in the integrated datasets combined TCGA with GTEx database. The analysis identified KLRB1 was significantly upregulated in tumor tissues with the corresponding normal tissues as controls (Fig. 1B, *P* < .05). We further evaluated the expression of KLRB1 in different pathological stages of patients with TGCT, in which KLRB1 was higher expressed in the stage1 than that of stage2/3 (Fig. 1C, *P* < .05). Besides, we compared the protein level of KLRB1 using the IHC staining by means of the THPA database. The protein expression level of KLRB1 in TGCT tumor tissues was higher than that in normal tissues (Fig. 1D). These findings suggested that the upregulation of KLRB1 in TGCT.

### 3.2. Expression of KLRB1 showed different clinicopathological features in TGCT

**Figure 2.**
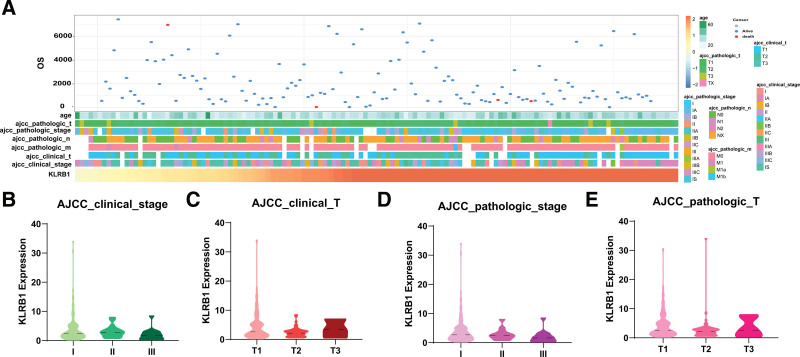
Association between KLRB1 and clinicopathological characteristics of gliomas. (A) The landscape of KLRB1-related clinicopathological features of gliomas in The Cancer Genome Atlas (TCGA) database. (B–E) Expression of KLRB1 in different clinical and pathological stages.

Patients with varying expression levels of KLRB1 showed distinct patterns of clinical and pathological characteristics. Clinical and pathologic data included age, American Joint Committee on Cancer (AJCC) pathologic tumor stage (TNM), AJCC clinical tumor stage, KLRB1 expression, and OS. The age, AJCC pathologic tumor stage (TNM), AJCC clinical tumor stage, and KLRB1 expression are presented as heat maps, while OS is presented as a dot plot in Figure 2A. Moreover, we further analyzed the expression of KLRB1 at different clinical and pathological stages in Figure 2B–E.

### 3.3. KLRB1 is a potential biomarker for the Seminoma

**Figure 3.**
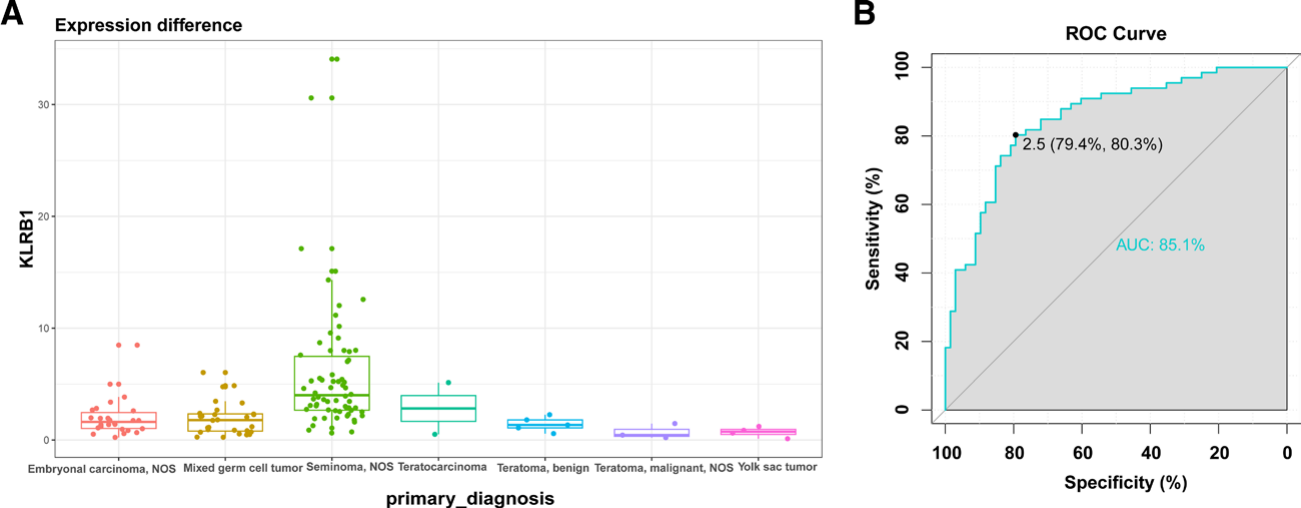
KLRB1 is specifically enriched in the seminoma type of TGCT. (A) KLRB1 was enriched in the seminoma type in The Cancer Genome Atlas (TCGA) databases. (B) The receiver-operating characteristic (ROC) curve showed the high-expression specificity of KLRB1in the seminoma type in the TCGA databases. AUC=area under the curve.

We explored the distribution of KLRB1 in different primary diagnosis types defined by the TCGA network. Compared with other TCGA primary diagnosis types, KLRB1 was significantly enriched in the Seminoma type in the TCGA databases (Fig. 3A). The receiver-operating characteristic curve was performed to evaluate the expression specificity of KLRB1 in the Seminoma type of TGCT. As expected, the area under the curve was up to 85.1% in the TCGA database (Fig. 3B). These results suggested that KLRB1 was explicitly enriched in the seminoma type and may function as a potential seminoma biomarker of TGCT.

### 3.4. KLRB1 is associated with immune response in TGCT

**Figure 4.**
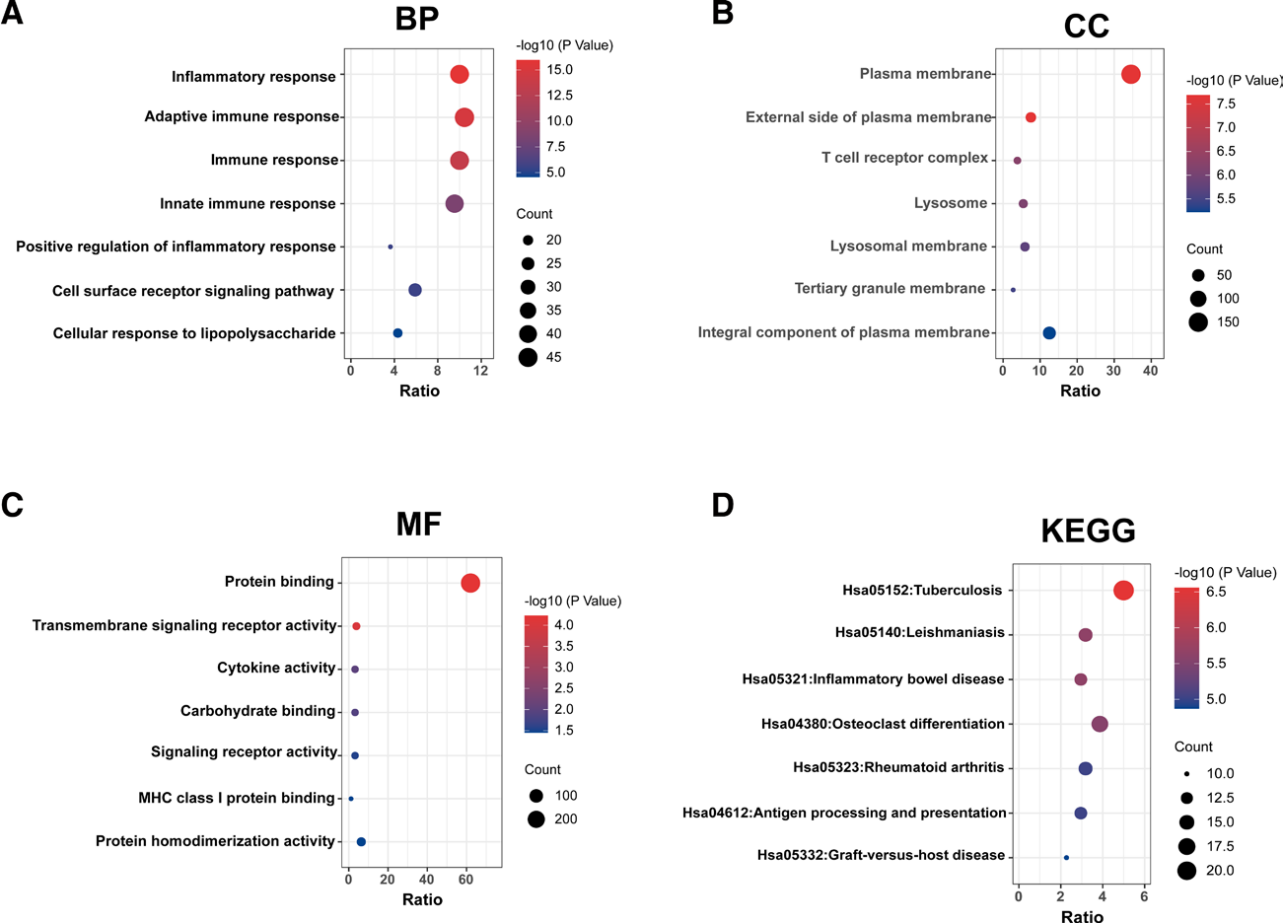
KLRB1 was closely related to immune response in TGCT. (A–C) Biological processes (BP), cellular components (CC), and molecular functions are mostly related to KLRB1 in the TCGA database. (D) Kyoto Encyclopedia of Genes and Genomes (KEGG) pathway analysis of KLRB1 in the TCGA database.

To explore the biological functions related to KLRB1, the genes most related to KLRB1 were screened out by Pearson correlation analysis (|R| > 0.5, *P* < .05) in the TCGA databases. GO and KEGG analysis based on the above gene sets were performed. In the TCGA database, biological processes most related to KLRB1 include immune response, adaptive immune response, and innate immune response (Fig. 4A). Moreover, KLRB1 most related cellular components were the plasma membrane (Fig. 4B). The molecular functions were protein binding (Fig. 4C). The signaling pathway most closely related to KLRB1 is tuberculosis, leishmaniasis, and inflammatory bowel disease pathway (Fig. 4D).

### 3.5. KLRB1 is highly positively correlated with the natural killer cells activation in the immune response

**Figure 5.**
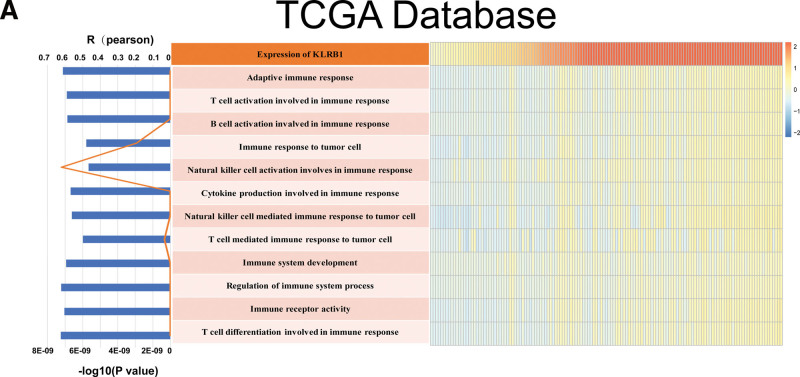
Correlation analysis between KLRB1 expression and immune function enrichment scores. (A) The heatmap showed the expression of KLRB1 and the enrichment scores of immune functions of each patient in The Cancer Genome Atlas (TCGA) databases. The samples were arranged in ascending order of the expression of KLRB1. The column graph and line graph on the right showed the *R*-value and *P*-value of the correlation analysis.

To further understand the involvement of KLRB1 in the process of tumor immunogenic cancer cell death, we tested the effect of KLRB1 activation on immune pathways and cytokine profiles. Gene set variation analysis (GSVA) in the TCGA database was used to determine enrichment scores for immune processes. Correlation analysis between enrichment scores and KLRB1 expression showed that KLRB1 expression was positively correlated with most immune functions, and it’s interesting that these results suggest that KLRB1 is closely positively associated with natural killer cell activation in the immune response in TGCT (Fig. 5A).

### 3.6. A positive association of KLRB1 with immune checkpoint inhibitors and impaired inflammatory regulation

**Figure 6.**
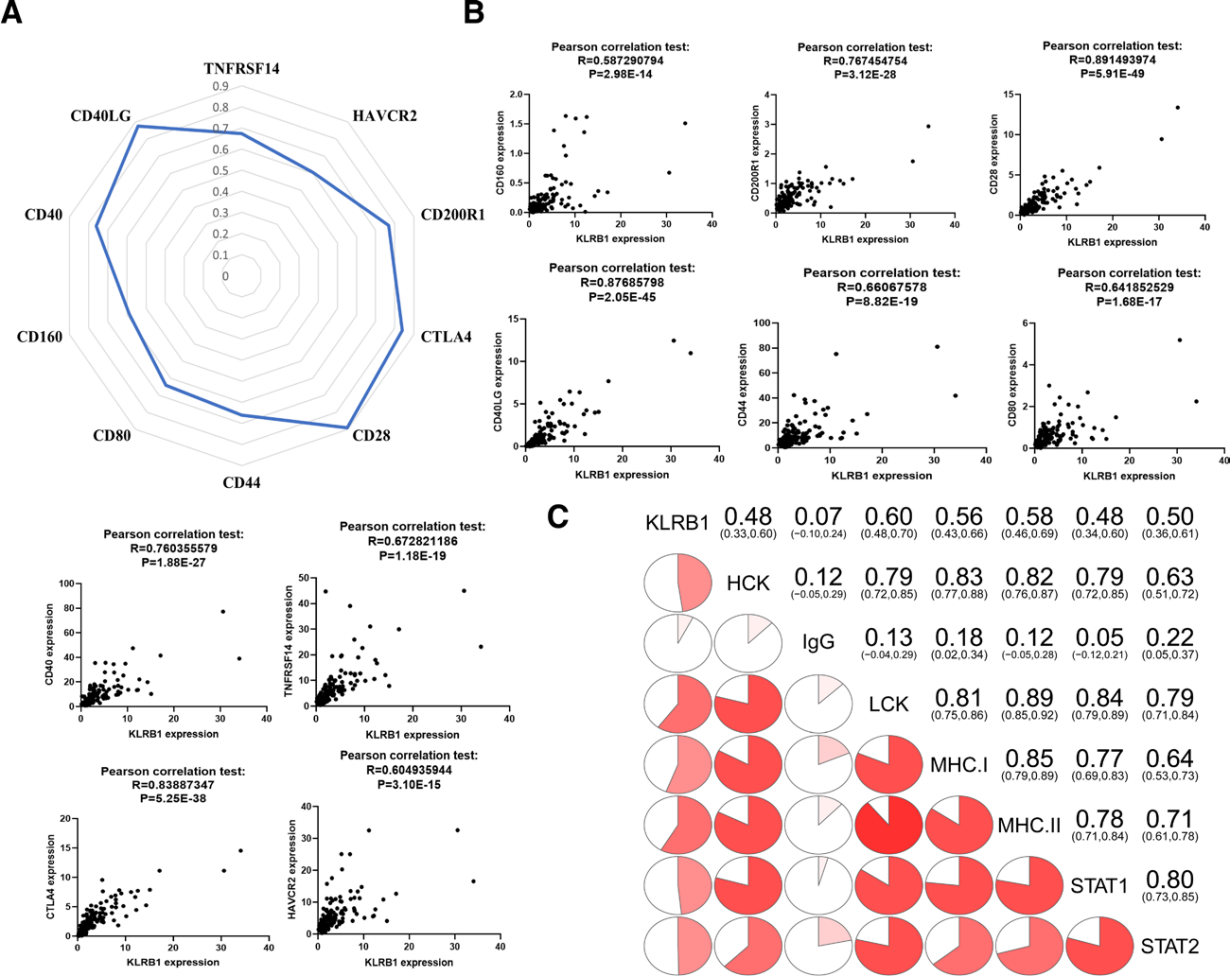
The correlation between KLRB1 expression and immune checkpoint inhibitors and inflammatory activities in The Cancer Genome Atlas (TCGA) databases. (A, B) Correlation analysis of KLRB1 expression levels with 10 common immune checkpoint gene levels in TGCT. (C) Correlation matrix between KLRB1 and inflammation-related metagenes. Correlation coefficients are shown in the upper right. Correlation coefficients are shown as a pie chart scale. The darker the color the stronger the correlation. Correlations were tested by Pearson correlation analysis.

ICIs work against tumors by blocking the binding of immune checkpoints to their ligands, thereby relieving the immune function suppression caused by immune checkpoints and reactivating immune cells.^[21,22]^ Subsequently, the correlation between KLRB1 expression and the expression of 10 common immune control genes was analyzed. Interestingly, in TGCT, KLRB1 expression was positively correlated with immune checkpoint markers, including CD40, CTLA4, CD44, CD28 and so on (Figure 6A, B). In addition, we selected 7 metagenomic clusters related to the immune system as markers of immune status,^[23,24]^ including hematopoietic cell kinase, lymphocyte-specific kinase (LCK), major histocompatibility complex, transcriptional signal transducer and activator 1/2 (STAT1/2), interferon, and IgG.^[25]^ Corrgrams showed that KLRB1 was significantly positively correlated with 7 metagenomic clusters in the TGCT database (Fig. 6C).

### 3.7. KLRB1 expression is correlated with immune-infiltration level

**Figure 7.**
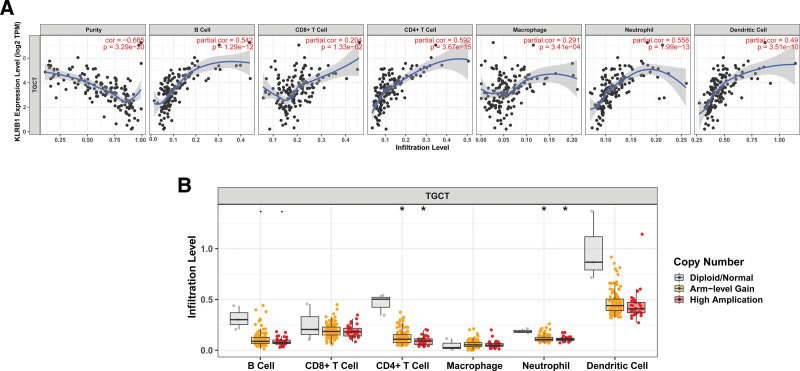
KLRB1 related immune infiltration analysis. (A) The correlation between KLRB1 and immune infiltration. (B) The relationship between KLRB1 copy number variation and the infiltration level of immune cell. *P < .05.

Tumor Immune Estimation Resource (TIMER) was employed to further investigate the immune infiltration of TGCT. The results showed that KLRB1 was negatively correlated with purity, but was positively correlated with B cell, CD8+ T-cell, CD4+ T-cell, Macrophage, Neutrophil, and Dendritic cell (Fig. 7A). In addition, the changes in the copy number of KLRB1 appeared to significantly affect the immune infiltration level in TGCT (Fig. 7B). Together, these results highlight that the alteration of KLRB1 copy number is closely related to the immune infiltration level. Thus, KLRB1 might be a potential reference value for determining the inhibition of TGCT progression and the development of new immunotherapies.

### 3.8. Construction of PPI and identification of hub genes

**Figure 8.**
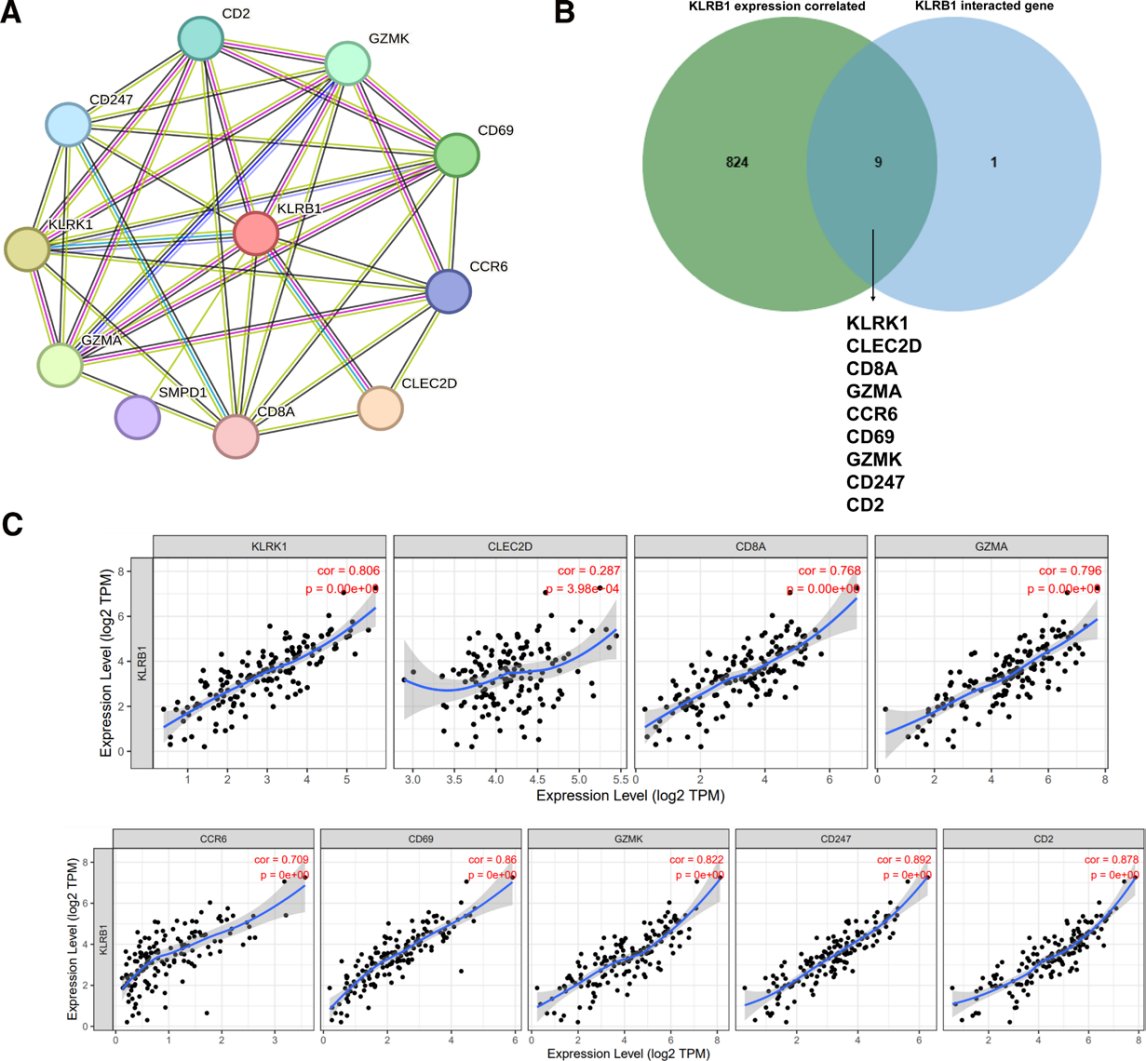
PPI network analysis of KLRB1-related genes. (A) The visualizing interaction network of KLRB1-binding proteins was obtained base on STRING database. (B) An intersection analysis of KLRB1 expression correlated genes and KLRB1-interacted genes was performed. (C) Correlation analysis between KLRB1 expression and screened common genes, including KLRK1, CLEC2D, CD8A, GZMA, CCR6, CD69, GZMK, CD247 and CD2.

To further investigate the internal mechanism of the KLRB1 gene in tumorigenesis, the PPI network analysis was performed by utilizing the STRING database. Figure 8A shows the visualizing interaction network of 11 KLRB1-binding proteins with the experimental evidence identification. In addition, through comparing KLRB1 expression correlated genes with KLRB1-interacted genes, we screened out the common members such as KLRB1, CLEC2D, CD8A, GZMA, CCR6, CD69, GZMK, CD247 and CD2 (Fig. 8B). Moreover, the KLRB1 expression level was remarkably positively correlated with that of KLRK1 (*R* = 0.806), CLEC2D (*R* = 0.287), CD8A (*R* = 0.768), GZMA (*R* = 0.796), CCR6 (*R* = 0.706), CD69 (*R* = 0.86), GZMK (*R* = 0.822), CD247 (*R* = 0.892) and CD2 (*R* = 0.878) (Fig. 8C).

## 4. Discussion

TGCT are a common malignancy occurring in young adult men.^[22]^ Despite the major treatment combining surgery with chemotherapy and radiotherapy being performed, there is still a depressing prognosis for TGCT patients.^[23,24]^ More and more studies have confirmed that TGCT have an important genetic component in their development, the majority of which are clustered in families.^[25]^ Approximately 50 susceptibility genes have been identified as polygenic genetic risks for TGCT.^[26]^ However, the functional roles of these genes in the development of TGCT are unclear, and the underlying molecular mechanisms are also complicated. Therefore, identifying and understanding more effective biomarkers is critical for treatment and prognosis prediction.

In recent years, cancer immunotherapy has provided a new modality for cancer treatment, and this means of treating certain cancers is a promising strategy that is gradually gaining acceptance.^[27]^ Currently, much of the information on immunotherapy for TGCT focuses on combinations of ICIs, vaccines, and other immunotherapeutic agents, radiotherapy, as well as radiochemotherapy or other molecularly targeted drugs.^[10,28]^ In particular, the major breakthrough in tumor immunotherapy was the discovery of immune checkpoint (IC) proteins, which led to the development of ICIs, which have emerged as a promising approach and have marked a paradigm shift in tumor immunotherapy. ICIs have provided long-term clinical benefits to a large number of patients suffering from a wide range of tumor types, including curing a subset of patients. Thus, the clinical success of ICIs has revolutionized the field of cancer immunotherapy.^[29,30]^

Recently, multiple reports suggest that the KLRB1 gene and the CD161 protein it encodes play a critical role in the development of tumorigenesis as well as in tumor immunity.^[11–13]^ Furthermore, inactivation of KLRB1 on T-cells enhanced the antitumor activity of tumor-infiltrating T-cells in vitro and in vivo, at least in part due to increased secretion of inflammatory cytokines.^[12,31]^ KLRB1 is a possible prognostic marker and therapeutic target linked with human esophageal squamous cell carcinoma^[17]^ and breast cancer tumors.^[32]^ In this study, we examined the expression of KLRB1 in diverse pathological types of TGCTs by analyzing mRNA sequence data from 134 TGCTs patients in the TCGA database. In our study, we showed that KLRB1 was upregulated in tumor tissues, especially in seminomas, and that elevated KLRB1 expression correlated with adverse clinicopathological features of TGCT. This provides insights into the expression and biological function of KLRB1 in TGCT, which suggests its potential function as an oncogene and prognostic biomarker.

To illustrate KLRB1 gene’s potential biological functions, we conducted GO and KEGG functional enrichment analyses, utilizing the top 500 genes most relevant to KLRB1. The results suggested that KLRB1 was mainly related to immune response and inflammatory response. Notably, KLRB1 has been correlated with T-cell response in glioblastoma and newly discovered as a suppressive immune checkpoint.^[18]^ The expression and regulation of KLRB1 define the effector CD4+ T-cells that respond quickly and are linked with better survival in tumors associated with HPV16.^[33]^ Tumor-infiltrating immune cells are critical elements of the tumor stroma and play a significant role in both tumor growth and sensitivity to cancer therapy.^[34]^ Our results also demonstrated that there is a robustly positive relationship between KLRB1 expression level and infiltration of macrophages, B cell, Dendritic cell, Neutrophil, CD4+ T-cells, and CD8+ T-cells, which indicated that KLRB1 may be involved in regulating tumor immunology in TGCTs.

We further explored the hub genes of KLRB1, and analyzed the 833 genes most associated with KLRB1 in the TGCA dataset and the genes most associated with KLRB1 in the Sting dataset in an interaction analysis to identify 9 genes (KLRK1, CLEC2D, CD8A, GZMA, CCR6, CD69, GZMK, CD247, and CD2). KLRK1, which encodes NKG2D, a homodimeric lectin-like receptor. Both KLRK1 and KLRB1 are expressed on natural killer cells and belong to the C-type lectin superfamily, and KLRK1 was identified as a prognostic biomarker for lung adenocarcinoma.^[35]^ In prostate cancer, the overexpression of CLEC2D on cancerous cells hinders NK cell-mediated killing by interacting with KLRB1.^[36]^ CD8a molecule (CD8A) may serve as an effective biosignature of the response to immunotherapy and infiltration of immune cells.^[37]^ Cytotoxic lymphocyte-derived granzyme A (GZMA) cleaves GSDMB, a protein of the gasdermin family that forms pores, leading to the onset of pyroptosis in the target cell.^[38]^ The CC chemokine receptor 6 (CCR6) is a G protein-coupled receptor (GPCR) implicated in various biological processes, and it is selectively expressed in immature dendritic cells and memory T-cells.^[39]^ CD69 as Potential Predictors of Responses to PD-1/PD-L1 Blockade Cancer Immunotherapy in Lung Cancer and Melanoma.^[40]^ GZMK shows promise as a potential diagnostic marker for the early detection of rheumatoid arthritis. This is especially true for patients who test negative for anti-citrullinated protein antibodies.^[41]^ The CD247 molecule, also known as T-cell surface glycoprotein CD3 zeta chain, has potential as a disease severity and prognostic biomarker for Idiopathic pulmonary fibrosis among T-cells.^[42]^ The glycoprotein CD2 is expressed primarily on T and NK cells and serves as a costimulatory receptor.^[43]^ In summary, these 9 genes are correlated with immune responses or with KLRB1, which requires further studies to explore the interaction of these genes with KLRB1 in TGCT.

In summary, our study demonstrated that KLRB1 was significantly highly expressed in TGCT, especially in seminomas. KLRB1 was highly correlated with natural killer cell activation in the immune response and correlated with the level of immune infiltration of tumor cells. This study is the first to investigate the potential biological function of KLRB1 in TGCT, which provides a new theoretical basis for the future treatment of TGCT. However, there are still some limitations in this study. it should be noted that only public databases (i.e., TCGA and the GTEx) were employed to analyze and confirm the relationship between KLRB1 expression and TGCT using bioinformatic methods, and our study lacks more in vivo and in vitro evidence. After we establish an appropriate design and make the appropriate preparations, we will conduct further research on the molecular functions and mechanisms of KLRB1 in TGCT through a series of cell-line, tissue, and animal experiments.

## Acknowledgments

We acknowledge the TCGA, GTEx, THPA, TIMER, GEPIA, and STRING databases for free use.

## Author contributions

**Conceptualization:** Luyu Li, Baojun Ju, Xiao Li.

**Data curation:** Yaorui Hu.

**Funding acquisition:** Baojun Ju.

**Investigation:** Yaorui Hu.

**Supervision:** Xiao Li.

**Writing—original draft**: Luyu Li, Yaorui Hu.

**Writing—review and editing:** Baojun Ju, Xiao Li.
